# Antiseptic Hairdressing

**Published:** 1903-07-04

**Authors:** 


### Antiseptic Hairdressing.

It is well known that a scratch or abrasion in the skin of a person employed in picking hair and sorting wool is liable to become infected with the germs of anthrax, and to develop into a malignant pustule. It is also well known that the poison of scarlet fever, now recognised as a germ disease, clings with great persistence to articles of clothing, *e.g.*, wool, hence the necessity of thorough disinfection of such materials, in connection with suppression of outbreaks of scarlet fever. The wool and hair serve as habitats for the germs of anthrax and scarlet fever, and it is no doubt true also that hair tends to support the germ-life of other infectious diseases which are assuredly passed on from one individual to another by contact with infected hair. And yet, while it can scarcely have failed to impress the mind of many intelligent and thoughtful persons that the condition of the modern hair-dressing establishment is such as to favour the wide distribution of infectious diseases, it is astounding how little attention has been directed to the question of improving the hygienic condition under which the toilet of the head and beard is publicly carried on.

Even in the so-called first-class establishments of our large cities, what are the conditions in the vast majority of instances to be found? Throughout a single day, many persons permit themselves to be shaved or to have their hair dressed, at the hands of a coiffeur, indifferent to the principles of asepsis. Be his customers the most immaculate of people in respect of cleanliness, there may yet be amongst them just one unsuspected "contact," unwittingly carrying the germs of an infectious disease, from whom, by the use of the comb, or hairbrush, perhaps shaving-brush, or razor, in common for a number of other customers through the day, the germs of infection may be widely disseminated. For to disinfect these instruments between use there is no attempt. And the hairdresser himself as little dreams of washing his hands or of disinfecting them between each "turn," as he does of sterilising his instruments. And yet he is permitted to rub his fingers into the scalp of first one person, and then into that of another, without any intervening process of cleansing them. Moreover, he will touch with his fingers, during the performance of his duties, any other article in his shop which he has occasion to require, so that not only other individuals, but the furniture, walls, etc., may become infected with germs, and serve as "contacts" for further distribution of infectious diseases, once the source has been introduced. The more abundant the hair, obviously the more potent the contact medium, so that the female attendant or the nurse, who, after caring for cases of scarlet fever, or of measles or other infections, carries out the process of disinfection of the hair imperfectly, proves a fruitful source of widespread infection, by the modern system of public hairdressing. And when we consider that such conditions are possible in the higher walks of life, what may we expect to find in the inferior barbers' shops scattered in their hundreds through our great metropolis, and throughout the country? In these latter the very conditions exist for spreading not only the specific infections, but such rebellious diseases as ringworm, many cases of baldness, sycosis, and favus.

Surely, then, it is high time that such an ordinance for the hygienic regulation of barbers' shops, as has been adopted by the Board of Health, Jersey City, should receive attention. That the time is ripe for an inquiry, with a view to possible legislation in this country, is also indicated by a movement in Germany and the United States in the same direction. Though the difficulties of carrying out in this country such Utopian schemes as are proposed elsewhere, may be found insuperable, yet much might be done by instituting a regular system of inspection of barbers' shops and insisting upon cleanliness and efficient ventilation; and more might be done throughout all classes of hairdressers' establishments by introducing appropriate methods of disinfecting hands and instruments immediately after use, as well as of destroying or of disinfecting the clippings of hair. The barber should observe scrupulous cleanliness regarding his own hands and person, by careful washing of hands immediately after attending to each case. Combs, brushes and other instruments should be properly sterilised by boiling or by immersion in formalin immediately after use, in short everything should be done to promote a system of aseptic hairdressing, and so prevent the spread of infectious diseases.

